# Supplementary material for: Neighborhood Environmental Factors and Physical Activity Status among Rural Older Adults in Japan
Source: Int J Environ Res Public Health. 2021 Feb 4;18(4):1450. doi: 10.3390/ijerph18041450 (PMC7913898; doi:10.3390/ijerph18041450)
Supplement: Supplementary file 1 [file ijerph-18-01450-s001.zip › Table_S1.pdf]

Table S1: Correlation between neighborhood environmental factors.

	**Slope**	**Distance to community center (CC)**	**Bus stop density**	**Intersection density**	**Residential density**
**Slope**	1	0.34	-0.4	-0.65	-0.49
**Distance to CC**	0.34	1	-0.41	-0.44	-0.35
**Bus stop density**	-0.4	-0.41	1	0.76	0.7
**Intersection density**	-0.65	-0.44	0.76	1	0.73
**Residential density**	-0.49	-0.35	0.7	0.73	1
