# Supplementary material for: Neighborhood Environmental Factors and Physical Activity Status among Rural Older Adults in Japan
Source: Int J Environ Res Public Health. 2021 Feb 4;18(4):1450. doi: 10.3390/ijerph18041450 (PMC7913898; doi:10.3390/ijerph18041450)
Supplement: Supplementary file 1 [file ijerph-18-01450-s001.zip › Table_S2.pdf]

Table S2: Cox proportional hazard model for physical inactivity by neighborhood environmental factors - stratified analysis by residential municipality and age group.

	**Unnan 60-74** n = 706	**Unnan 75+** n = 257	**Oki 60-74** n = 365	**Oki 75+** n = 203	**Onan 60-74** n = 680
	**HR (95%CI)**	**HR (95%CI)**	**HR (95%CI)**	**HR (95%CI)**	**HR (95%CI)**
**Slope (ref. 1st, lowest)**					
2nd	1.17	1.30	1.55*	1.89	0.88
	(0.89, 1.46)	(0.76, 1.85)	(1.08, 2.02)	(0.82, 2.97)	(0.57, 1.20)
3rd	0.88	1.27	1.74*	1.61	1.18
	(0.60, 1.17)	(0.72, 1.81)	(1.27, 2.21)	(0.58, 2.65)	(0.86, 1.50)
4th	0.91	1.49	0.89	3.13*	0.82
(highest)	(0.60, 1.23)	(0.99, 1.99)	(0.27, 1.50)	(2.09, 4.16)	(0.53, 1.10)
**Distance to community center (ref. 1st, lowest)**					
2nd	1.17	0.55	1.09	0.48	0.94
	(0.89, 1.45)	(0.03, 1.06)	(0.59, 1.60)	(-0.58, 1.54)	(0.70, 1.26)
3rd	0.95	0.69	1.06	0.52	0.95
	(0.66, 1.23)	(0.25, 1.14)	(0.50, 1.62)	(-0.60, 1.64)	(0.70, 1.27)
4th	0.92	0.72	1.03	0.73	0.66*
(highest)	(0.60, 1.25)	(0.17, 1.27)	(0.55, 1.51)	(-0.08, 1.54)	(0.47, 0.93)

Note: All models for each municipality and age group adjusted for sex, smoking drinking, BMI, musculoskeletal disorders. *p<0.05. There were no subjects >= 75 years old in Onan.
