# Supplementary material for: Neighborhood Environmental Factors and Physical Activity Status among Rural Older Adults in Japan
Source: Int J Environ Res Public Health. 2021 Feb 4;18(4):1450. doi: 10.3390/ijerph18041450 (PMC7913898; doi:10.3390/ijerph18041450)
Supplement: Supplementary file 1 [file ijerph-18-01450-s001.zip › Figure_S1_5.pdf]

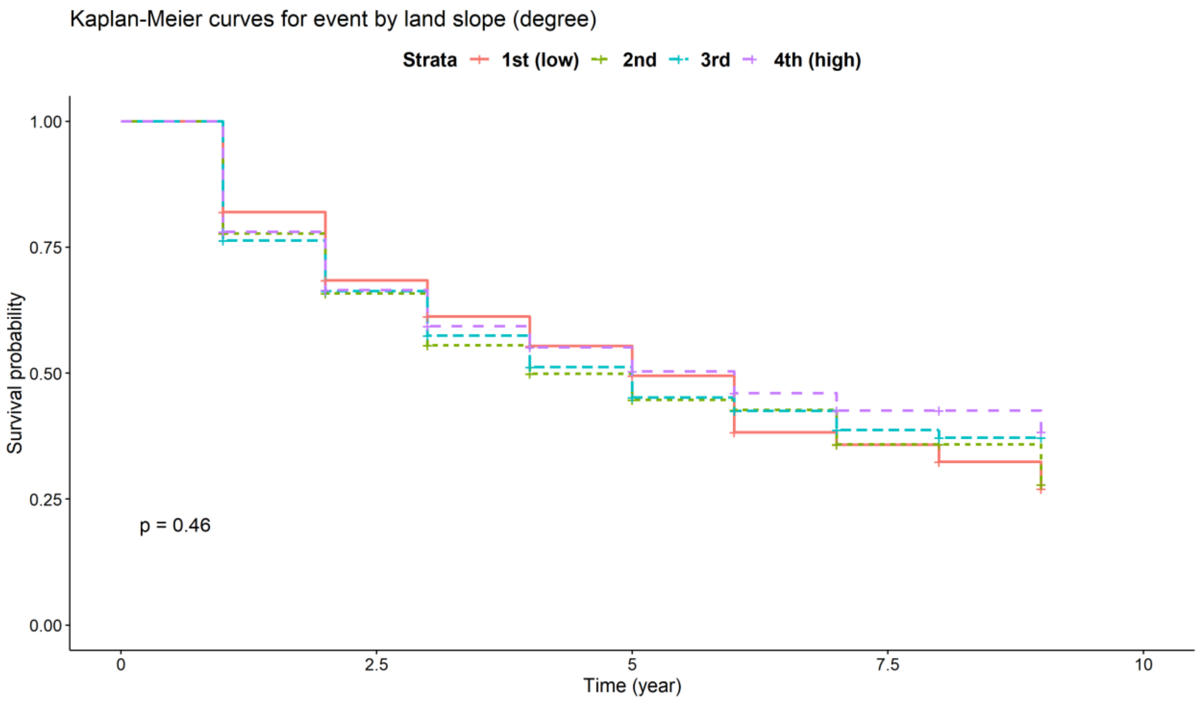

Figure S1. Kaplan-Meir curves for the time until becoming physically inactive by quartile category of land slope (degree). Quartile 1 (1st) = 3.41, 6.98; Quartile 2 (2nd) = 6.98, 10.1; Quartile 3 (3rd) = 10.1, 13.7; Quartile 4 (4th) = 13.7, 26.2

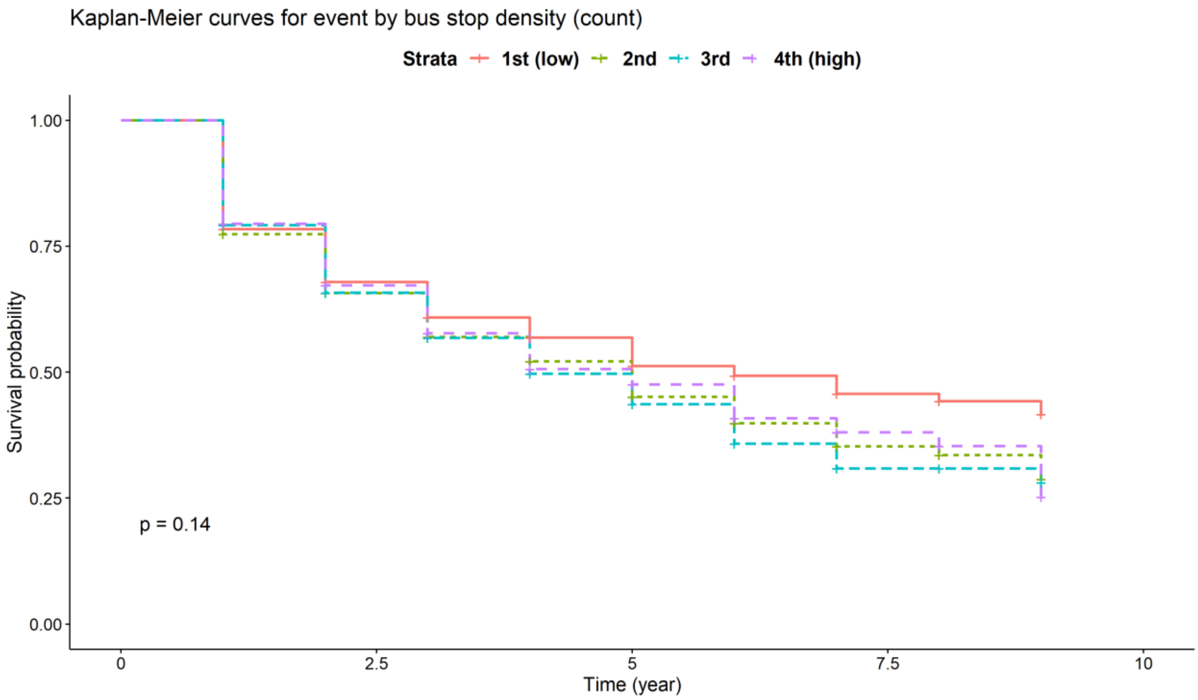

Figure S2. Kaplan-Meir curves for the time until becoming physically inactive by quartile category of bus stop density (count). Quartile 1 (lowest) = 0, 2; Quartile 2 (low) = 2, 4; Quartile 3 (high) = 4, 7; Quartile 4 (highest) = 7, 27

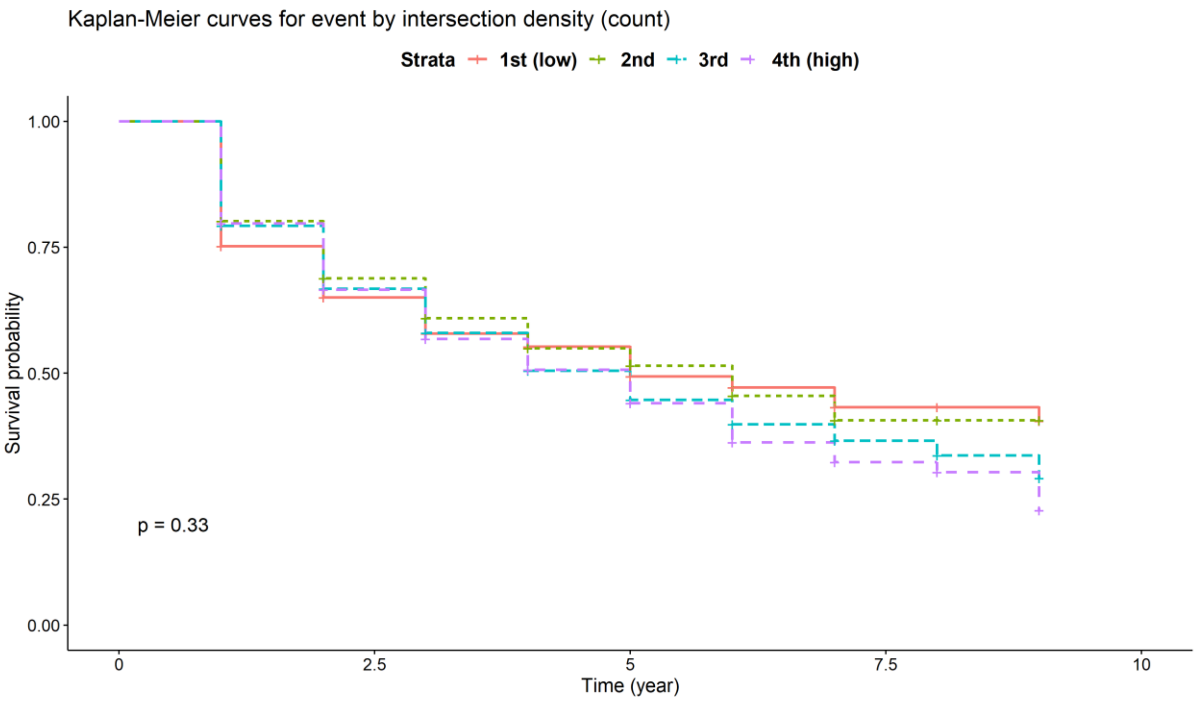

Figure S3. Kaplan-Meir curves for the time until becoming physically inactive by quartile category of intersection density (count). Quartile 1 (lowest) = 0, 9; Quartile 2 (low) = 9, 20; Quartile 3 (high) = 20, 40; Quartile 4 (highest) = 40, 126

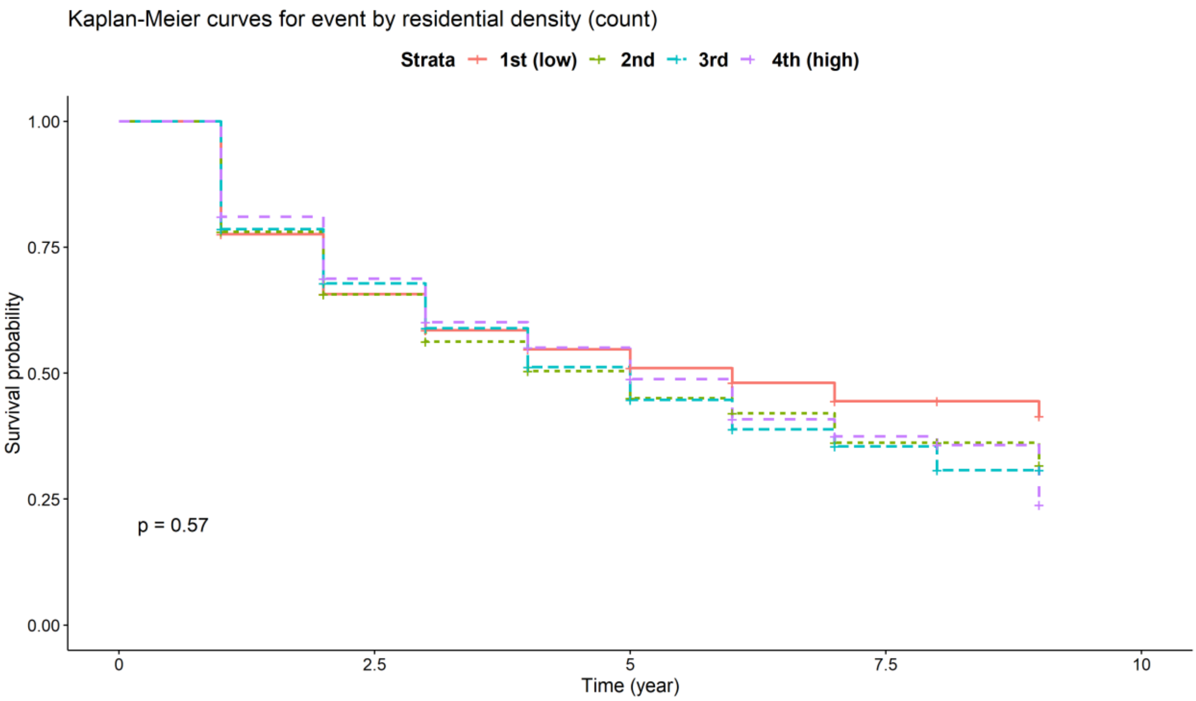

Figure S4. Kaplan-Meir curves for the time until becoming physically inactive by quartile category of residential density (count). Quartile 1 (lowest) = 0; Quartile 2 (low) = 1, 42; Quartile 3 (high) = 42, 166; Quartile 4 (highest) = 166, 1607

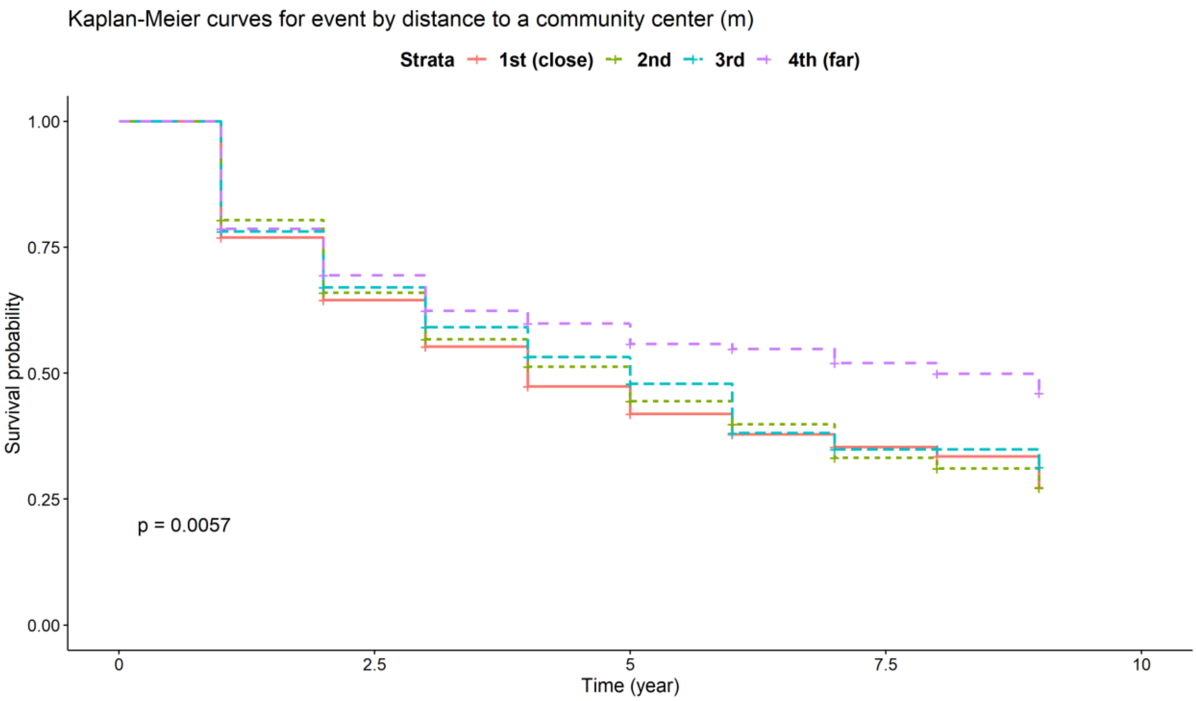

Figure S5. Kaplan-Meir curves for the time until becoming physically inactive by quartile category of distance to a community center (m). Quartile 1 (closest) = 0.97, 835; Quartile 2 (close) = 835, 1540; Quartile 3 (far) = 1.540, 3.030; Quartile 4 (farthest) = 3030,11200
