# Supplementary figures and images for: Neighborhood Environmental Factors and Physical Activity Status among Rural Older Adults in Japan
Source: Int J Environ Res Public Health. 2021 Feb 4;18(4):1450. doi: 10.3390/ijerph18041450 (PMC7913898; doi:10.3390/ijerph18041450)

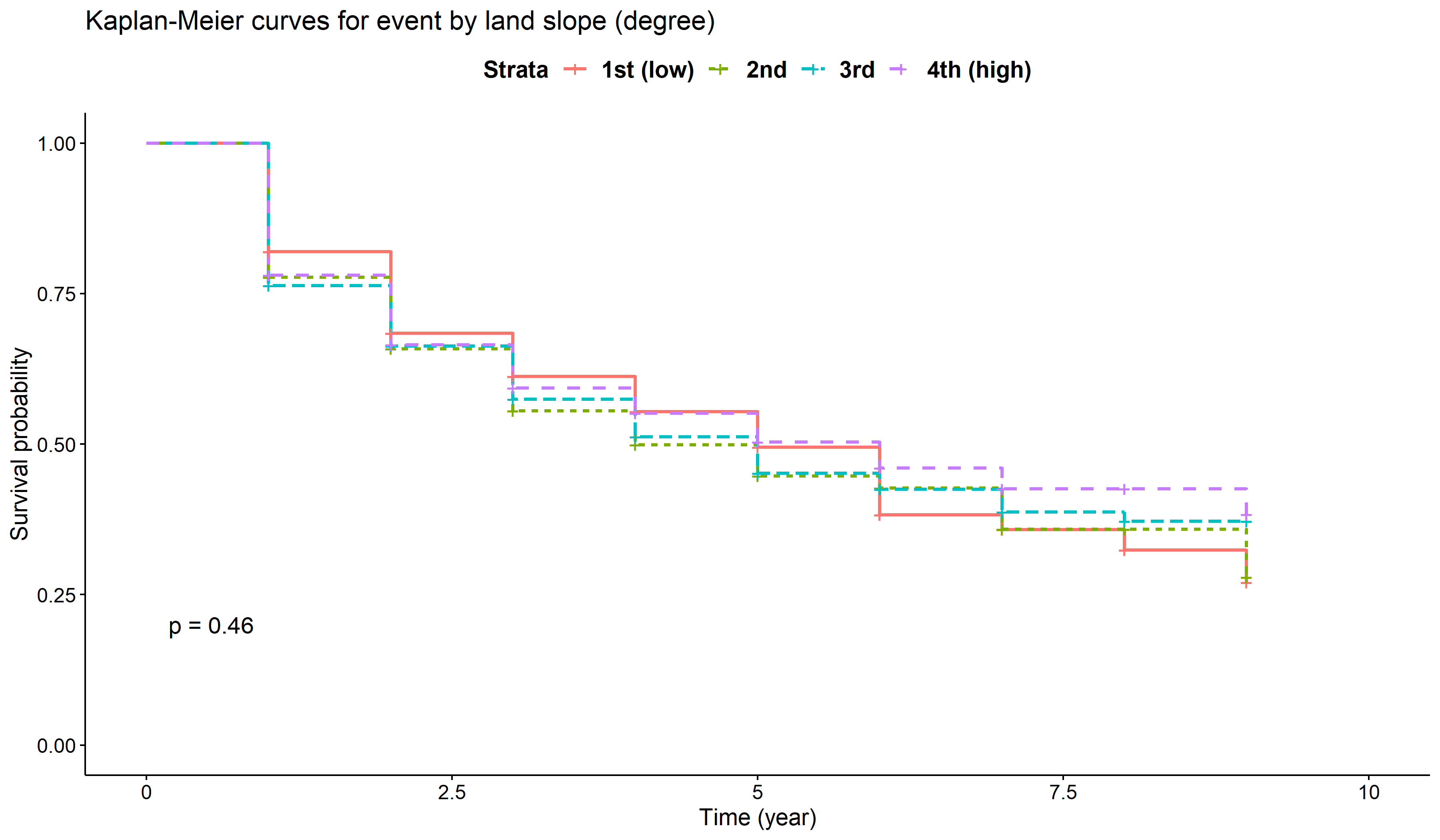

Supplement: Supplementary file 1 [file ijerph-18-01450-s001.zip › Figure_S1.png]

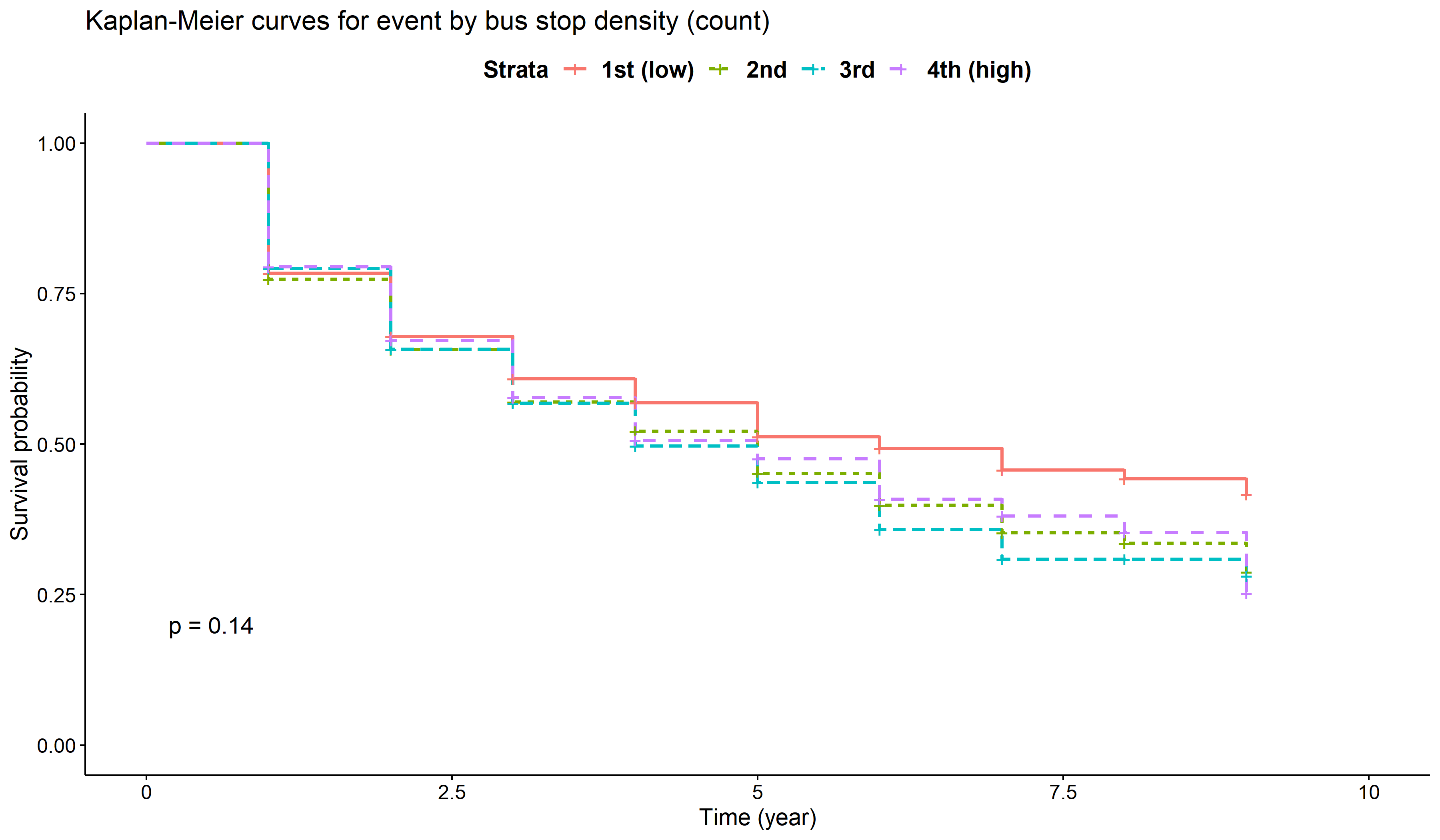

Supplement: Supplementary file 1 [file ijerph-18-01450-s001.zip › Figure_S2.png]

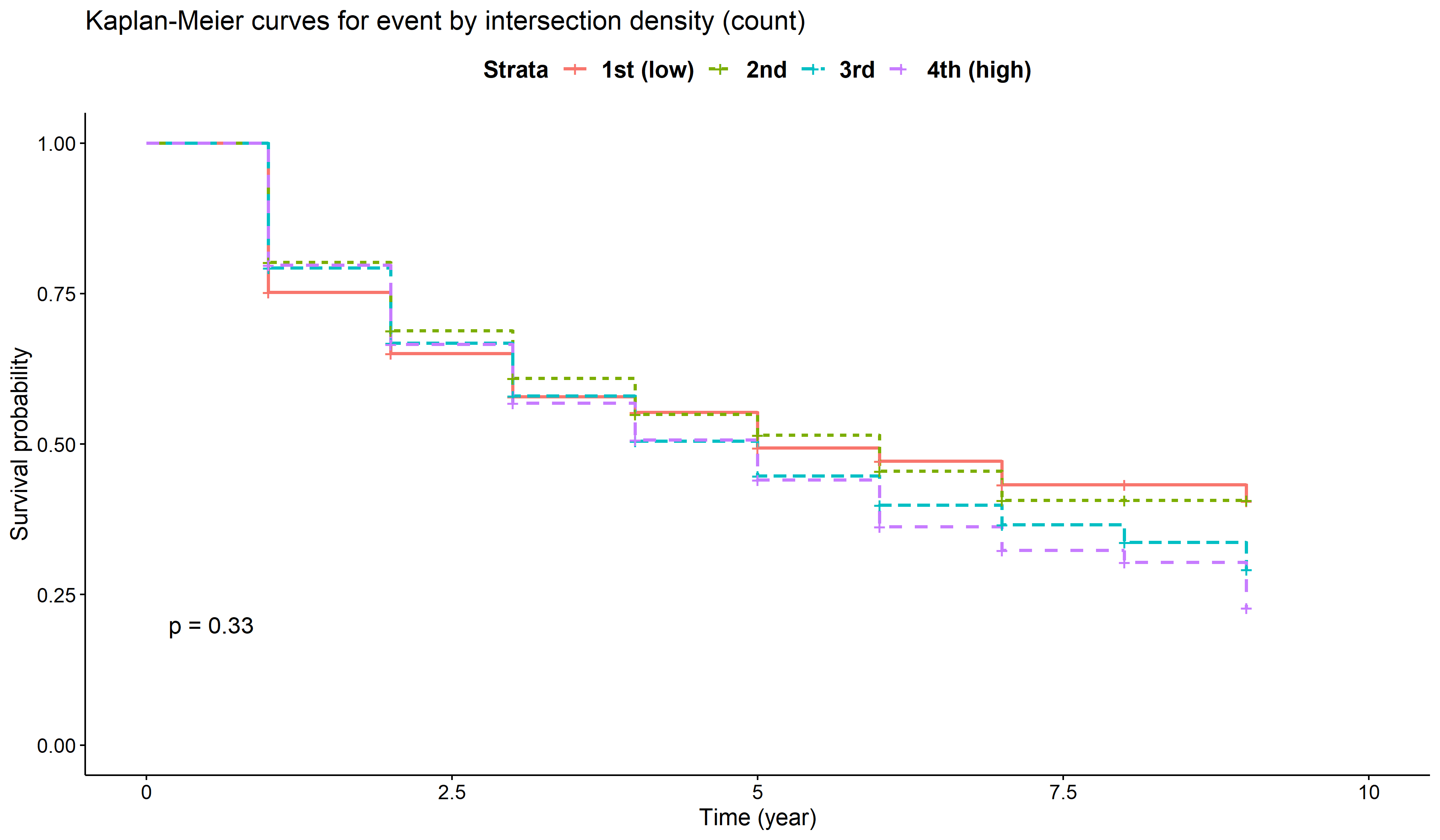

Supplement: Supplementary file 1 [file ijerph-18-01450-s001.zip › Figure_S3.png]

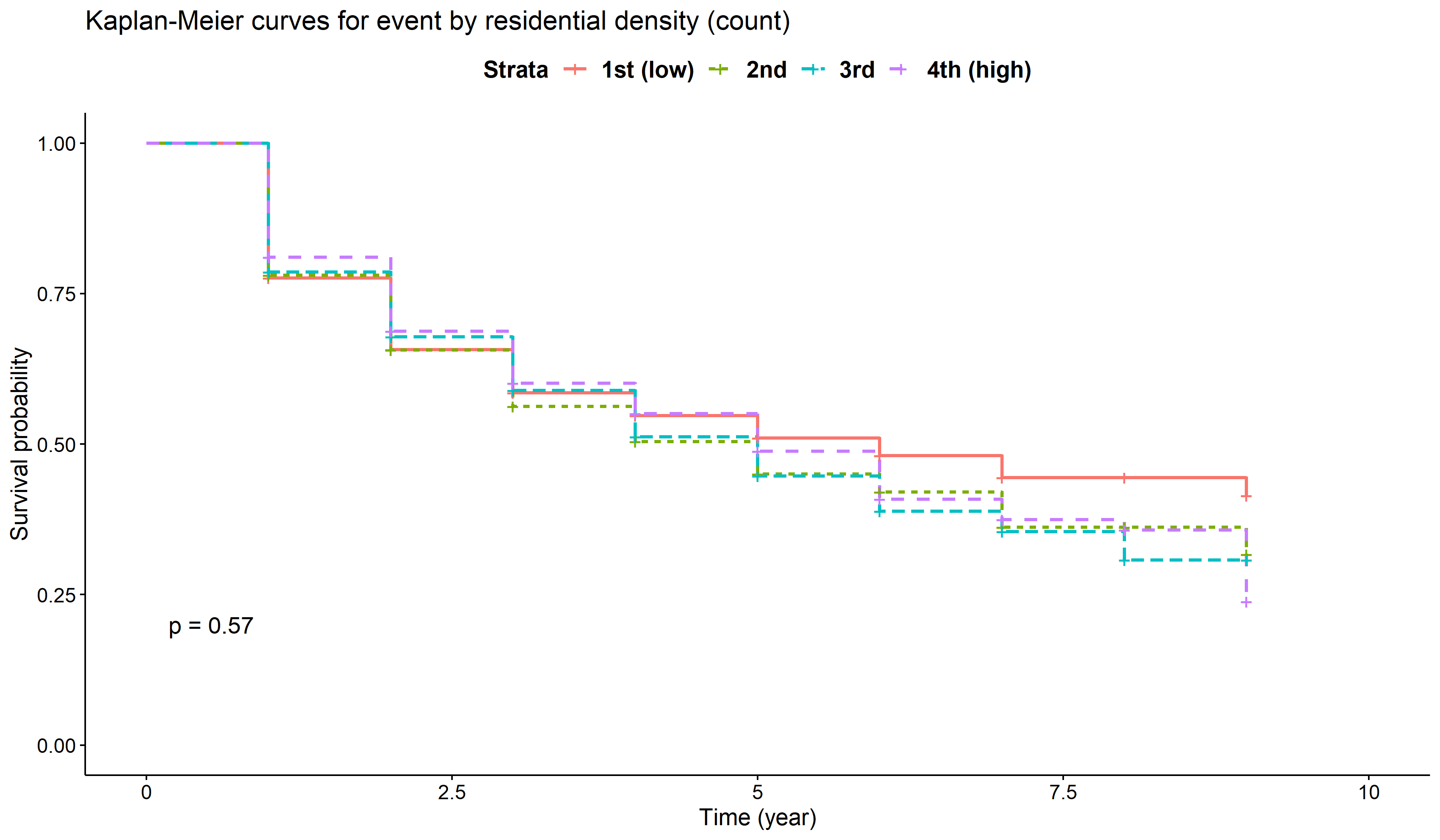

Supplement: Supplementary file 1 [file ijerph-18-01450-s001.zip › Figure_S4.png]

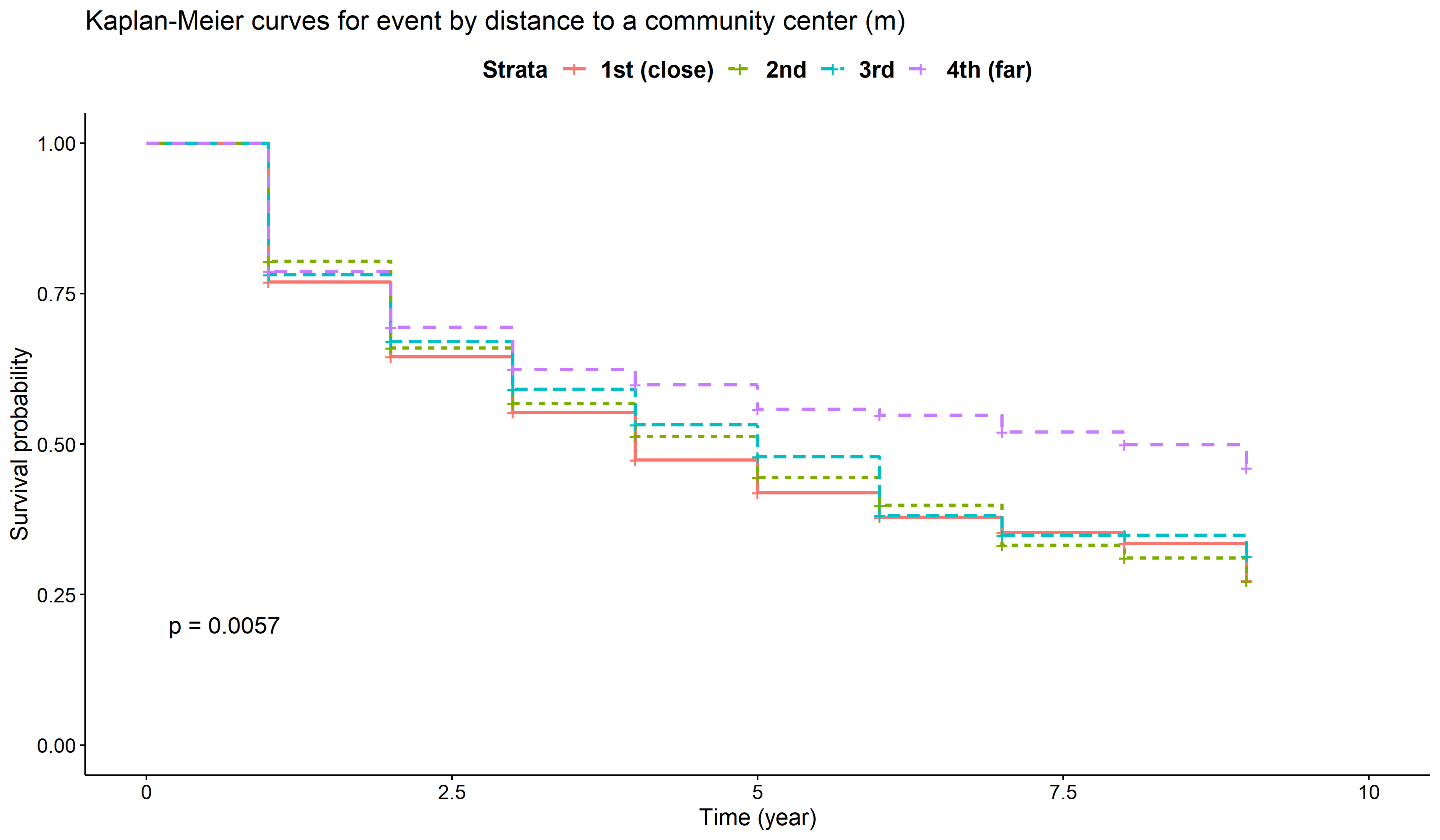

Supplement: Supplementary file 1 [file ijerph-18-01450-s001.zip › Figure_S5.png]
